# Pig Weight Estimation Method Based on a Framework Combining Mask R-CNN and Ensemble Regression Model

**DOI:** 10.3390/ani14142122

**Published:** 2024-07-20

**Authors:** Sheng Jiang, Guoxu Zhang, Zhencai Shen, Ping Zhong, Junyan Tan, Jianfeng Liu

**Affiliations:** 1 College of Science, China Agricultural University, Beijing 100083, China; shengjiang@cau.edu.cn (S.J.); zhencai688@sina.com (Z.S.); zping@cau.edu.cn (P.Z.); 2 National Innovation Center for Digital Fishery, China Agricultural University, Beijing 100083, China; zgx985211@cau.edu.cn; 3 College of Information and Electrical Engineering, China Agricultural University, Beijing 100083, China; 4 Key Laboratory of Agricultural Information Acquisition, Ministry of Agriculture, Beijing 100083, China; 5 Beijing Engineering and Technology Research Center for Internet of Things in Agriculture, Beijing 100083, China; 6 College of Animal Science and Technology, China Agricultural University, Beijing 100083, China

**Keywords:** deep learning, deep information, attitude correction, live weight estimation of pigs

## Abstract

**Simple Summary:**

Welfare farming of pigs is a kind of farming method that pays attention to the welfare of animals, which is of great significance to the health and production performance of pigs. The estimation of pig weight based on computer vision can avoid direct contact with pigs, reduce the loss caused by contact or pressure, and ultimately improve the overall breeding efficiency and economic benefits. In this paper, we proposed a new pig weight estimation method based on Mask R-CNN and machine learning methods. Our new method extracted features from a new perspective to solve the problem of illumination and body bending.The experimental results show that our method can accurately predict the weight of pigs.

**Abstract:**

Using computer vision technology to estimate pig live weight is an important method to realize pig welfare. But there are two key issues that affect pigs’ weight estimation: one is the uneven illumination, which leads to unclear contour extraction of pigs, and the other is the bending of the pig body, which leads to incorrect pig body information. For the first one, Mask R-CNN was used to extract the contour of the pig, and the obtained mask image was converted into a binary image from which we were able to obtain a more accurate contour image. For the second one, the body length, hip width and the distance from the camera to the pig back were corrected by XGBoost and actual measured information. Then we analyzed the rationality of the extracted features. Three feature combination strategies were used to predict pig weight. In total, 1505 back images of 39 pigs obtained using Azure kinect DK were used in the numerical experiments. The highest prediction accuracy is XGBoost, with an MAE of 0.389, RMSE of 0.576, MAPE of 0.318% and $R^{2}$ of 0.995. We also recommend using the Mask R-CNN + RFR method because it has fairly high precision in each strategy. The experimental results show that our proposed method has excellent performance in live weight estimation of pigs.

## 1. Introduction

Pigs are one of the most important animals in the world, and their meat plays an important role in people’s daily consumption and nutritional needs [1,2]. At present, many countries are encouraging pig welfare farming in which the breeding goal is respecting and improving the wellbeing of pigs [3]. Pig welfare farming emphasizes addressing the behavioral, physiological and psychological needs of pigs, and it also focuses on providing an appropriate environment, feeding conditions and management measures to improve the health and quality of pigs’ lives.

Currently, more and more animals are slaughtered each year for food production. The growing market demand for animal products has promoted the development of intensive animal husbandry, and motivated farmers to increase the number of animals in the herds without more resources. To meet the market demand and take good care of each animal, automated tools become a good choice [4,5].

During the breeding process, weight is an important index to measure the growth and development of pigs [6]. Regularly recording pigs’ weights is helpful to understand their growth progress, health status and development. In addition, it is also helpful to judge the feeding effect and quality, so that the farmer can timely adjust feeding management and then improve breeding efficiency [7]. In terms of marketing, pig weight is an important indicator for buyers and sellers to evaluate the quality and value of pigs.

At present, the pig weight measurement methods include direct and indirect methods. The direct method is that the employees of the pig farm directly rush the pigs to the scale for weighing [8,9]. Although this is probably the most accurate measurement, it requires a lot of manpower and time, and also leads to the cost increase of pig breeding [10,11]. Furthermore, excessive manual contact may induce the spread of swine fever in pigs, affecting the health of pigs and the quality of pork.

With the development of computer vision (CV), automatic non-contact pig weight estimation has attracted more attention [12,13]. Computer vision-based pig weight estimation can reduce the pressure of manual intervention and the labor costs, so it can promote healthy development of farms [14]. The key steps of computer vision-based pig weight estimation are as follows. Firstly, the contours of a pig’s back are obtained by image processing. Secondly, features are extracted based on the obtained contours, including body length and width of the pig, and the perimeter and area of the pig’s back in the vertical view image [15]. Finally, body weight is estimated according to the obtained features.

In the image processing stage, the core problem is how to segment the contours of the animal in the complex livestock environment. The initial segmentation is based on the threshold method. All pixels are divided into object and background according to the gray value and threshold. Although the threshold method is straightforward to implement, its effectiveness is often limited. Then, graph partitioning is developed to solve the segmentation with complex scenes [16,17,18]. However, graph partitioning easily leads to blurred boundaries, and performs poor on the noise or complex texture images. With the development of deep learning, object detection methods have been applied to extract pig contours [19]. Cang et al. designed an improved Faster R-CNN to estimate the weight of the pig by vertical view images, which integrated pig detection and the live weight regression branch into an end-to-end network [20]. Tengtrairat et al. extracted the contours of tilapia by three object detection methods (Faster R-CNN, RetinaNet, and YOLO) as well as the instance segmentation method Mask R-CNN [21]. They concluded that Mask R-CNN had the highest accuracy in extracting the contours.

Since Mask R-CNN uses the RoI Align process, it can select the high-level features. It outputs not only the bounding box, but also the pixel-level mask of objects. The features of each position can be extracted more accurately, and it avoids the problem of feature deviation and can improve the accuracy. In addition, Mask R-CNN is faster, more adaptable and more flexible. So, we adopt Mask R-CNN to extract the contours of pig’s back, and focused on solving the problems of contour error caused by illumination and noise.

In order to estimate the live weight accurately, the proper features should be extracted. The most commonly used features are body length and body width. In addition, the perimeter and area of the pig’s back image, eccentricity, axis length, body height and volume may also be useful [22,23]. Pezzuolo et al. acquired the top-down and side-looking depth images of live pigs, extracted the features of body length, heart perimeter and body height, and then built a multiple quadratic regression model with a goodness of fit of 0.9942 and an MAE of 0.48 kg [9]. Subsequently, Zhang et al. used a multi-output regression convolutional neural network to estimate the weight and size of the pigs by the shoulder width, shoulder height, hip width, hip height and body length. The correlation coefficient between the estimated results and the actual results was between 0.9879 and 0.9973 [24]. Li et al. used a single frame surface point cloud to measure the body length, body height and body width of pigs and built a model to estimate the weight of pigs [25].

Although there are many features that have been extracted for weight prediction, image-based feature information remains to be mined. In addition, the pig body image is curved in some images, so if the features extracted from the images are used directly for weight prediction, there will be prediction errors. So how to correct the features is also a challenge.

Regarding the difficult issues mentioned above, this paper proposed a new pig weight estimation method that combines Mask R-CNN and ensemble regressors. The innovations of this paper are as below:

(1) Mask R-CNN was used to extract the contours of the pig accurately by using RoI Align better. We converted the resulting image into a binary image. It solved the problem of uneven illumination on the image.

(2) We extracted the body size information and image features, and used the actual measured body size information to correct the body length and hip width. Furthermore, the depth information, which is the height from the camera to the pig back, was also used as a feature, which solved the problem of body bending. Finally, the rationality of the features was analyzed.

(3) A large number of regressors and deep learning methods were used to make predictions on three different kinds of features.

## 2. Materials and Methods

### 2.1. Abbreviated Specification

First, we introduced the definition of abbreviations and acronyms used in the article as shown in Table 1.

### 2.2. Experimental Data

#### 2.2.1. Data Acquisition

The data used in this paper were collected from 27 February to 2 March 2023 from a commercial farm in Shao Guan, Guangdong Province, China. Before collecting the images of pigs, a total of 39 white York pigs’ body length, body width, hip width, body height, hip height and weight were measured. To ensure methodical consistency, all measurements were taken by the same person and repeated three times; the average value was recorded as the index value of each pig. The details are shown in Table 2.

**Figure 1:**
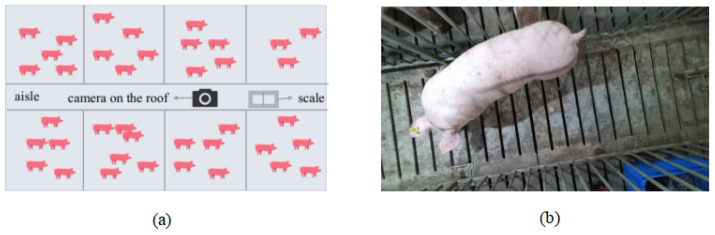
(**a**) Bottom view of the piggery. (**b**) Representative camera view of the pigsty.

The image data was collected using the Azure DK camera with image resolution 1920 × 1080. The camera was fixed at 145 cm above the scale, and aligned with the center line of the channel. It was pointed straight down during the image acquisition process. The pig farm has a solid concrete floor. After the pig enters the channel, close the entrance and exit of the channel to ensure that there is only one pig in the channel. Let the pigs naturally pass through the recording range and then obtain the back image of each pig. A representative camera view of the pigsty is shown in Figure 1.

#### 2.2.2. Data Set Construction and Annotation Procedures

**Figure 2:**
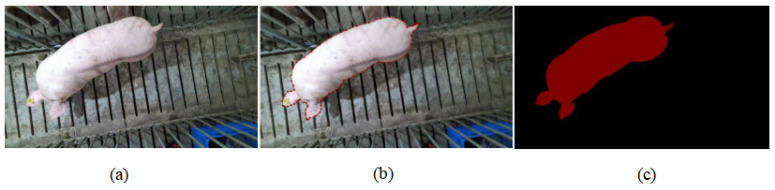
(**a**) Image that needs to be labeled. (**b**) Image after labeling (**a**) with labelme. (**c**) Mask image generated after annotation.

For the captured pig videos, we used Python 3.7 language to write scripts, segmented the 39 recorded videos frame by frame, and collected a total of 1505 pig pictures. The tagged process was performed with labelme tool (https://github.com/wkentaro/labelme/tree/v3.11.2). Figure 2 shows an example of an input image and the output of the annotation process.

### 2.3. The Proposed Methodology

**Figure 3:**
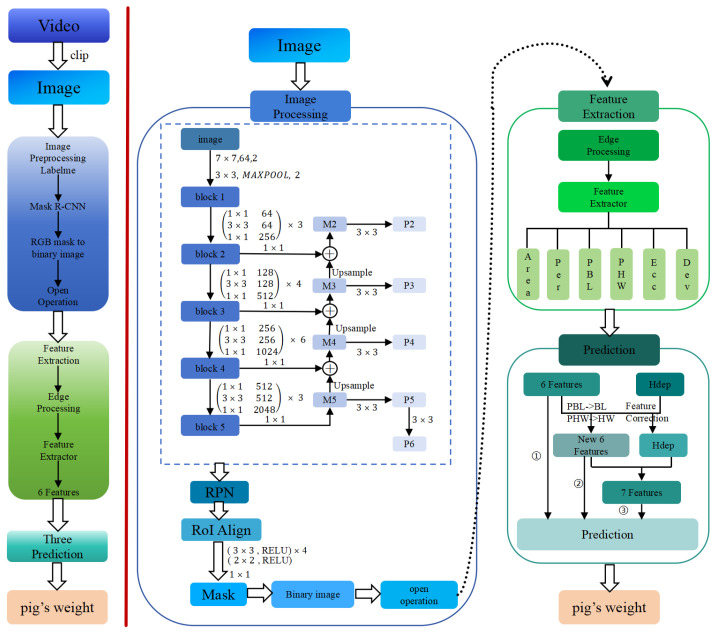
The frame diagram of the method. The left of the figure is the overall architecture of the proposed approach. It mainly includes three steps: image processing, feature extraction and weight prediction. The right of figure is the detailed steps of the method. In the image processing stage, we used Mask R-CNN to extract the contours of the pig. Then we transformed the mask image into a binary image, and performed an open operation on the image. In the feature extraction stage, the edge of the processed image was extracted first, and then the feature extractor was used to extract the feature. In the weight prediction stage, we used three different strategies to predict weight. Firstly, we used image features to estimate weight directly. Secondly, we used quadratic corrected features to estimate weight. Finally, we added depth information as features to estimate weight.

In this section, we describe our method. Firstly, we introduce the image processing stage, then feature processing and model training are inducted. The principle of our method is shown in Figure 3.

#### 2.3.1. Image Processing

Mask R-CNN was used to process the image. The Mask R-CNN frame shown in Figure 3 is an object detection and instance segmentation algorithm based on deep learning [26]. First, it uses a multi-scale enhanced feature pyramid structure to extract the feature map of the image, and the generated feature map then passes through region proposal networks (RPNs) to generate a series of candidate target boxes. RoI Align converts candidate boxes of different sizes into fixed-size feature blocks, which are used for mask generation.

Mask R-CNN needs to be trained with a bounding box and mask for each target. It trains the network using multitask loss functions, including target classification loss, bounding box regression loss and mask generation loss. Its loss function is:

(1) $$Loss=L_{class}+L_{BB}+L_{mask}$$

where $L_{class}$, $L_{BB}$, $L_{mask}$ represent classification loss, boundary box loss, and average binary cross entropy loss.

$L_{class}$ combines RPN and Mask R-CNN classification loss during head training. $L_{BB}$ demonstrate the model’s positioning effect on objects. $L_{class}$, $L_{BB}$ are calculated by Formulas (2) and (3):

(2) $$L_{class}\left(p,u\right)=-logP_{u}$$

where $L_{class}\left(p,u\right)$ is the predicted probability of ground truth class *u* for each positive bounding box.

(3) $$L_{BB}\left(t^{u},v\right)=\sum ie\left\{x,y,w,h\right\}\left[L_{1}^{smooth}\left(t^{u}-v_{i}\right)\right]$$

where $L^{smooth}\left(x\right)=\left\{\begin{array}{cc} 0.5x^{2}, & \mathrm{if}\;|x|<1\\ |x|-0.5, & \mathrm{otherwise} \end{array}\right.$, and $L_{1}^{smooth}\left(t^{u}-v_{i}\right)$ represents the predicted bounding box for class *u* and ground truth bounding box *v* for each input *i*.

The $L_{mask}$ has *m* dimensional output for each RoI, where *K* represents a number of a class and *m* is a matrix representation of the class. A per-pixel sigmoid is applied, and the $L_{mask}$ is computed using the average binary cross-entropy loss that the *K* mask is associated with the $K_{th}$ class, i.e., K=1=pig. The $L_{mask}$ can be expressed in Equation (4):

(4) $$L_{mask}=\frac{1}{m^{2}}\sum_{1}^{m^{2}}\left(logP_{i,j}^{k}\right)$$

where $P_{i,j}^{k}$ denotes the $i_{th}$ pixel of the $j_{th}$ generated mask.

**Figure 4:**
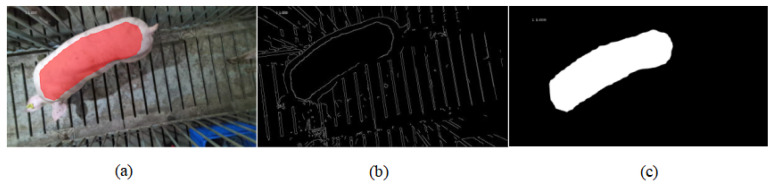
(**a**) Mask image. (**b**) The image after edge extraction operation of (**a**). (**c**) The image that converts (**a**) to a binary image.

After Mask R-CNN, the colored mask images were obtained. But color information is affected by illumination and color deviation, which usually generate noise interference. The conversion to binary images can reduce this interference and focuses more on the texture and shape features of the target object. Therefore, we converted the obtained mask images into binary images. After obtaining the binary images, we used the ellipse check of size 70 to carry out morphological processing to remove the noise. Figure 4 is the comparison between the edge extraction of the mask image and the corresponding binary image.

#### 2.3.2. Feature Extraction

The direct and indirect features were used for prediction. These are the area and the perimeter of the back image, the pixel value of body length and hip width, the eccentricity of the fitted ellipse and the deviation of the image.

(1) The area (Area) of the back image. We overlay the foreground pixels in a binary image on a grid and treat each foreground pixel as a square with a side length equal to the length of the grid [27]. The area of the entire binary image is the sum of the areas of the square corresponding to all foreground pixels.

(2) The perimeter (Per) of the back image. We calculate Per by the edge contours of the image. Each line segment is the distance between two adjacent boundary points, and the perimeter is the sum of the lengths of all line segments.

(3) The pixel value of body length (PBL) and the pixel value of hip width (PHW). The minimum external moment of the mask area is calculated, and the length and width of the minimum external moment are BL and HW.

(4) The eccentricity of the mask region (Ecc). We perform elliptic fitting on the mask region, and obtain the major axis length and minor axis length of the fitting ellipse, then calculate Ecc.

(5) The deviation (Dev) of the binary image, which is firstly extracted for weight prediction. By calculating Dev, the proportion of the mask image in the whole image can be just reflected, and then some image depth information can be obtained.

Now, we have six features: Area, Per, PBL, PHW, Ecc and Dev. Since body bending may lead to the disturbance of PBL and PHW, they need to be corrected. Furthermore, the distance from the camera to the pig back ($H_{\mathrm{dep}}$) may have an impact on body size, which will affect the estimation of weight. So, $H_{\mathrm{dep}}$ should be estimated. Note that, the actual $H_{\mathrm{dep}}$ is calculate by

(5) $$H_{\mathrm{dep}}=H_{g}-\frac{\left(H_{b}+H_{h}\right)}{2}$$

where $H_{g}$, $H_{b}$, $H_{h}$ are actual measured and represent the distance of the camera from the ground, and the body height and the hip height, respectively. Then, PBL, PHW, Dev and $H_{\mathrm{dep}}$ are used as the input, and the actual body length and hip width are the output. We construct XGBoost to estimate BL, HW and $H_{\mathrm{dep}}$. The estimated values of XGBoost are used as new features, i.e., we have seven features: Area, Per, BL, HW, Ecc, Dev and $H_{\mathrm{dep}}$.

#### 2.3.3. Weight Estimation

In our study, four ensemble learning methods were used, including Adaboost, random forest regression (RFR), XGBoost and Stacking. Some regression methods were also used, including Kernel Ridge Regression (KRR), Support vector regression (SVR), Lasso regression (Lasso), Linear regression (LR), and deep learning methods such as BP neural network (BP) and Multilayer perceptron (MLP). Random forest regression is an ensemble learning method that combines multiple decision trees for classification or regression. We use information gain to select the best partitioning features. The formula is as follows:

(6) $$Gain\left(D,A\right)=Ent\left(D\right)-\sum_{v=1}^{V}\frac{|D^{v}|}{\left|D\right|}\cdot Ent\left(D^{v}\right)$$

where *D* is the data set, *A* is feature *A* in the feature set, *v* is the number of values of feature *A*, $D^{v}$ is the subset of samples whose value of feature *A* is *v*, and Ent(D) represents the information entropy of data set *D*.

Each decision tree is trained on randomly selected sample subsets and feature subsets, and finally determines the prediction result of the whole forest through voting or averaging. The regression prediction formula of random forest is as follows:

(7) $$\hat{Y}=\frac{1}{N}\sum_{i=1}^{N}f_{i}\left(X\right)$$

where $\hat{Y}$ is the predicted output of the model, *N* is the number of decision trees in the random forest, $f_{i}\left(X\right)$ is the prediction output of the *i*th decision tree, and *X* is the input feature.

XGBoost (eXtreme Gradient Boosting) is a machine learning algorithm based on gradient tree, which is widely used in regression tasks. It is extended and optimized on the basis of gradient lifting tree, and has excellent results and high-speed computing performance. The objective function of XGBoost includes the loss function and the regularization term, and its algorithm can be expressed in the following form:

(8) $$Obj\left(\theta \right)=\sum_{i=1}^{n}L\left(y_{i},{\hat{y}}_{i}\right)+\sum_{k=1}^{K}\Omega \left(f_{k}\right)$$

where θ represents the parameters of the entire model, $L\left(y_{i},{\hat{y}}_{i}\right)=\frac{1}{2}{\left(y_{i}-{\hat{y}}_{i}\right)}^{2}$ is a loss function, $f_{k}$ is the $k_{th}$ tree, $\Omega \left(f\right)=\gamma T+\frac{1}{2}{\lambda ||w||}^{2}$ is a regularization term, $y_{i}$ is the real label for sample *i*, ${\hat{y}}_{i}$ is the predicted label of sample *i*, *T* is the number of leaf nodes, *w* is the weight of the leaf node, and γ and λ are regularization parameters that control the weight of the regularization term.

Three different strategies were used to predict the weight of pigs, and all of our data processing was performed through python software.

In the first strategy, the features extracted from images were used, i.e.,:

$\left\{X_{k}=\left({\mathrm{Area}}_{k},{\mathrm{Per}}_{k},{\mathrm{PBL}}_{k},{\mathrm{PHW}}_{k},{\mathrm{Ecc}}_{k},{\mathrm{Dev}}_{k}\right),Y_{k}=W_{k}\right\},k=1,2,\ldots ,1505$. In order to verify the effectiveness of the new feature Dev, we also used the first five features without Dev for prediction.

In the second strategy, the corrected BL and HW were used to solve the problem of body bending. Thus, the data set in the second strategy was:

$\left\{X_{k}=\left({\mathrm{Area}}_{k},{\mathrm{Per}}_{k},{\mathrm{BL}}_{k},{\mathrm{HW}}_{k},{\mathrm{Ecc}}_{k},{\mathrm{Dev}}_{k}\right),Y_{k}=W_{k}\right\},k=1,2,\ldots ,1505$. We used the features to re-predict the weight of the pig according to the method in strategy 1.

In the third strategy, we took $H_{\mathrm{dep}}$ as a new feature to strategy 2 (3), so the dataset was $\{X_{k}=\left({\mathrm{Area}}_{k},{\mathrm{Per}}_{k},{\mathrm{BL}}_{k},{\mathrm{HW}}_{k},{\mathrm{Ecc}}_{k},{\mathrm{Dev}}_{k},H_{{\mathrm{dep}}_{k}}\right),Y_{k}=W_{k}\},k=1,2,\ldots ,1505$. Then we re-predicted the weight of the pig according to the method in strategy 1.

### 2.4. Implementation Details

The experimental hardware environment was equipped with a NVIDIA GeForce RTX 3090 Ti GPU with 24 GB of RAM, 2.20 GHz Intel Xeon Silver 4114 cpu, and a total of 128 GB of RAM. The operating system was an instance of Ubuntu 20.04.1, all running on a 64-bit system. A CUDA Toolkit 11.7 was installed with CUDNN 8.4.1, Python 3.7.0 and Tensorflow 1.13.1 with CUDA version 11.6. The automatic screening program was used to divide the data set into training sets and test sets, the ratio of which was 7:3. The training set was used in the training of the model, and the test set was used to evaluate the trained model. Network search and five-fold cross-validation were used to optimize the model parameters. For Mask R-CNN in our test, we used resnet50 as the backbone network. The learning rate, batch size and epoch were set to 0.001, 64 and 400, respectively.

### 2.5. Evaluation Metrics

We measured regression error using the mean absolute error (MAE), which is commonly used in regression models and reflects the actual error. The MAE is calculated as follows:

(9) $$MAE=\frac{1}{M}\sum_{i=1}^{M}\left|{\hat{y}}_{i}-y_{i}\right|$$

where $y_{i}$ is the true value of the predicted sample and ${\hat{y}}_{i}$ is the predicted value.

In addition to MAE, we also used mean absolute percentage error (MAPE), root mean square error (RMSE) and coefficient of determination ($R^{2}$) as evaluation indicators. MAPE, RMSE and $R^{2}$ are calculated as follow:

(10) $$MAPE=\frac{100\%}{M}\sum_{i=1}^{M}\left|\frac{{\hat{y}}_{i}-y_{i}}{y_{i}}\right|$$

(11) $$RMSE=\sqrt{\frac{1}{M}\sum_{i=1}^{M}{\left({\hat{y}}_{i}-y_{i}\right)}^{2}}$$

(12) $$R^{2}=1-\frac{\sum_{i=1}^{M}{\left({\hat{y}}_{i}-y_{i}\right)}^{2}}{\sum_{i=1}^{M}{\left({\bar{y}}_{i}-y_{i}\right)}^{2}}$$

where ${\bar{y}}_{i}$ is the average of the predicted values.

## 3. Experiment Results and Analysis

### 3.1. Results and Discussion

#### 3.1.1. Correlation Analysis between Different Features and Weight

**Figure 5:**
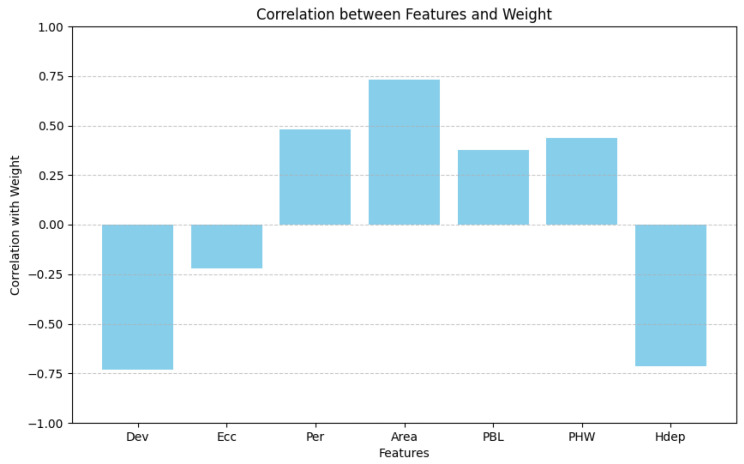
The Pearson correlation coefficient between different features and body weight. Dev is a deviation of the image. Ecc is the eccentricity of the fitted ellipse on the image. Per is the perimeter of the mask image. Area is the area of the mask image. PBL is the pixel value of the pig’s body length. PHW is the pixel value of the pig’s hip width.

First, we calculated the Pearson correlation coefficient between different features and weight. The results are shown in Figure 5. It can be seen that Area, Per, PBL and PHW are positively correlated with the weight of pigs, and Area has the highest correlation with the weight, which is in line with the physiological science of breeding pigs. Ecc and Dev are negatively correlated with the weight of pigs, which is also reasonable. When we collected data in the pig farm, we find that, when a pig is relatively thin and long, its weight will be relatively lighter than a short but thick pig. We think that this is directly related to the chest size of the breeding pig. Therefore, when the Ecc is large, it reflects the fact that the breeding pig is slender and the weight of the pig will be relatively small. Similarly, we can learn from the deviation formula (4) that, the greater the Dev is, the greater the proportion of black pixels there is in the binary image and correspondingly fewer white pixels, which indicates the greater distance from the camera. Because the camera is fixed on the ceiling, when the distance between the pig’s back and the camera is farther, it means that the pig is relatively small and its weight is relatively small. We can see that $H_{\mathrm{dep}}$ has high correlation with the weight, indicating that our substitution is successful and the information that we mine is very effective.

#### 3.1.2. Predicted Results of Pig Weight

**Figure 6:**
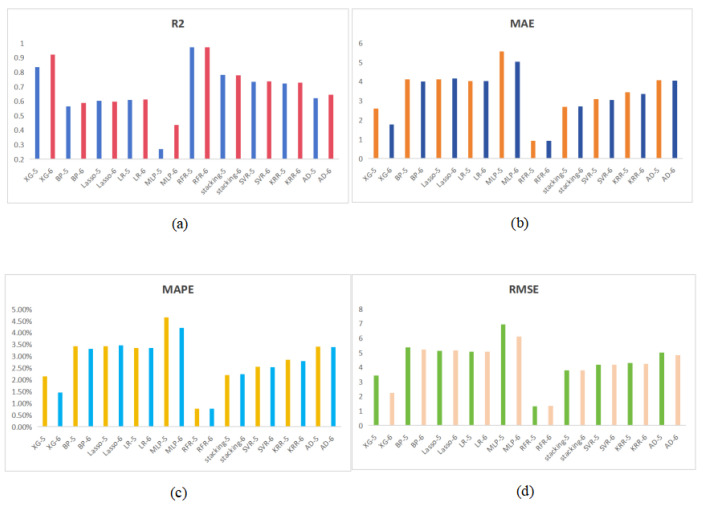
The calculation results of 10 kinds of models. (**a**–**d**) represent the result comparison graphs of MAE, MAPE and RMSE.-5 indicates the results of weight prediction using only the five features of Area, Per, PBL, PHW and Ecc.-6 indicates the results after adding the feature Dev.

The model in the first strategy was realized by learning the six features of the mask image: Area, Per, PBL, PHW, Ecc and Dev. The results obtained are shown in Table 3. Based on the values in Table 3, the RFR model provides the best results among 10 training algorithms. The MAE, RMSE, MAPE and $R^{2}$ values are 0.932 kg, 1.353 kg, 0.775% and 0.972, respectively. In order to prove the effectiveness of our image feature extraction, we also used the five features of Area, Per, PBL, PHW and Ecc to carry out the weight estimation of pigs. The results obtained are shown in Table 4. We present the two results in a bar chart (Figure 6). We can see that, when the feature Dev is added, the weight estimation is more accurate and the correlation coefficient is also improved. So Dev is a key feature to predict pigs’ weight.

**Figure 7:**
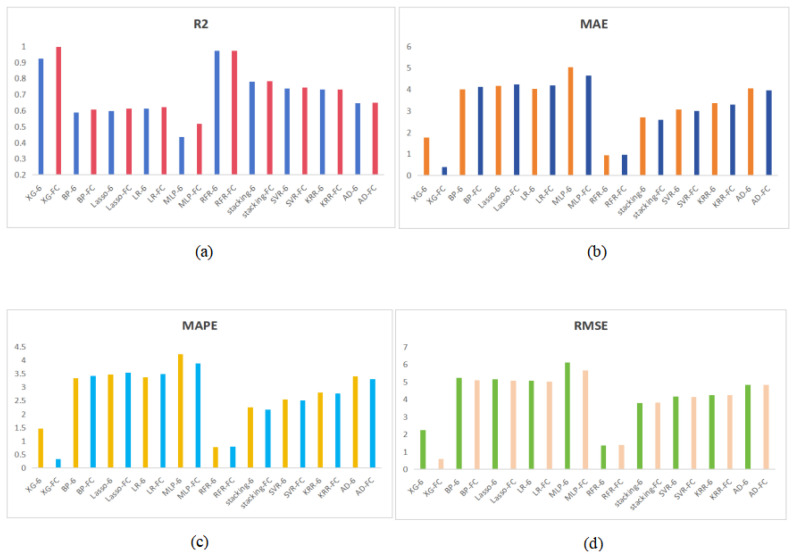
The calculation results of 10 kinds of models. (**a**–**d**) represent the result comparison graphs of MAE, MAPE and RMSE.-6 indicates the results of weight prediction using the features of Area, Per, PBL, PHW, Ecc and Dev.-FC indicates the results after feature correction.

In the second strategy, the predicted BL and HW replaced PBL and PHW. When we predicted BL, the MAE, RMSE, MAPE and $R^{2}$ values were 0.872 cm, 2.935 cm, 1.550% and 0.999, respectively. When HW was predicted, the MAE, RMSE, MAPE and $R^{2}$ values were 0.889 cm, 0.796 cm, 1.701% and 0.999, respectively. Because of the high accuracy, the predicted BL and HW were very close to the actual value. The weight prediction results on new features are shown in Table 5. Among them, the most accurate method is XGBoost, with an MAE of 0.389, RMSE of 0.576, MAPE of 0.318% and $R^{2}$ of 0.995. We present the results of Table 3 and Table 5 in the form of bar charts. As can be seen from Figure 7, our feature correction is successful. This also means that we have solved the problem of pig bending.

In the third strategy, we add $H_{\mathrm{dep}}$ as a new feature to corrected features. The results are shown in Table 6. We find that the method with the highest accuracy is random forest regression (RFR), with an MAE of 0.895 RMSE of 1.300, MAPE of 0.745% and $R^{2}$ of 0.974. Except for the XGBoost method, all the other methods have significantly improved the prediction accuracy.

As can be seen from Table 3, Table 5 and Table 6, when the data and features are small, the effect of using deep learning methods to determine pig weights is not good. Deep learning will have better effects when dealing with large amounts of data. This paper only has a data set of 39 pigs, with a small number of pictures and files, so deep learning is not applicable.

**Figure 8:**
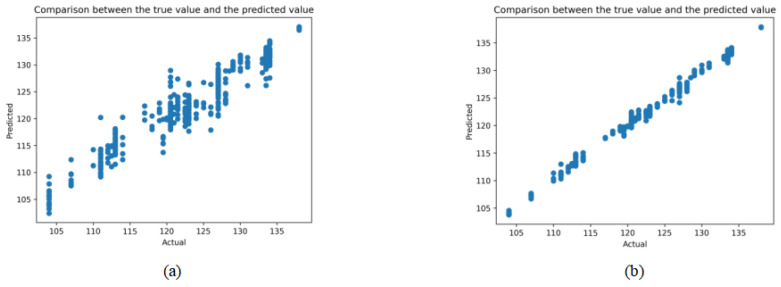
Results between the predicted value and the true value of the model before and after feature correction. (**a**) Results of the XGBoost method. (**b**) Results of the XGBoost method after feature correction.

We compare the graph between the predicted value and the true value of the model before and after feature correction. The method we used was XGBoost. The result is shown in Figure 8. We can see that, after feature correction, the prediction accuracy of the model is significantly improved.

Some typical examples of previous work are listed in Table 7, and we can see that our method has obtained very good results.

## 4. Conclusions

This paper proposed a new pig weight estimation method based on a deep learning and regression method. In total, 1505 back images of 39 pigs obtained by Azure kinect DK were used in the numerical experiments. To solve the problem of uneven illumination, the mask images were converted to binary images so that the edges and details of the target object were more clear. In order to solve the problem of the pig body bending, we extracted the image feature Dev and corrected PBL, PHW and Hdep using actual information and XGBoost. In addition, we analyzed the rationality of the extracted features. In the model stage, 10 machine learning methods in total were used to estimate the live weight of pigs in three different strategies. All three strategies provide good predictions of body weight. The one with the highest prediction accuracy is XGBoost, with an MAE of 0.389, RMSE of 0.576, MAPE of 0.318% and $R^{2}$ of 0.995. The results show that our method is accurate and reliable in weight estimation. Furthermore, the combination of Mask R-CNN and the ensemble regression method XGBoost is the best one in our article. We also recommend using the Mask R-CNN + RFR method because it has fairly high precision in each strategy.

We also need to restrict the movement of the pigs in our experiments. We have requirements in the shooting site that the pigs pass through the corridor one at a time. At the same time, we do not take full advantage of the side-looking information we collect from the pigs. Our method is only suitable for fattening pigs weighing 100–150 kg, and models and parameters need to be re-tested in other weight ranges. In the next stage, we plan to design a model to test the weight of pigs in all weight ranges and focus on the above issues.

## Figures and Tables

**Table 1:** Abbreviations appearing in the article as well as their full names.

Name	Abbreviation	Name	Abbreviation
Extreme Gradient Boosting	XGBoost	The area of the mask image	Area
Computer vision	CV	Deviation of the image	Dev
Random forest regression	RFR	The distance from the camera to the pig back	Hdep
Kernel Ridge Regression	KRR	Standard deviation	SD
Support vector regression	SVR	Minimum value	MIN
Lasso regression	Lasso	Maximum value	MAX
Linear regression	LR	Body length	BL
Multilayer perceptron	MLP	Hip width	HW
BP neural network	BP	Mean absolute error	MAE
The pixel value of the pig’s body length	PBL	Mean absolute percentage error	MAPE
The pixel value of the pig’s hip width	PHW	Root mean square error	RMSE
The eccentricity of the fitted ellipse on the image	Ecc	Coefficient of determination	R2
The perimeter of the mask image	Per		

**Table 2:** The trait, number of pigs, mean value, standard deviation (SD), minimum value (MIN) and maximum value (MAX).

Trait	Number	Mean	SD	MIN	MAX
Weight	39	122.81	7.86	104	138
Body Length	39	126	5.79	108	137
Hip Width	39	34.28	1.72	30	38
Body Height	39	69.4	2.00	65	74
Hip Height	39	75.13	2.55	70	82

**Table 3:** The weight estimation results of strategy 1.

Method	XGBoost	BP	Lasso	LR	MLP	RFR	Stacking	SVR	KRR	Adaboost
$R^{2}$	0.921	0.587	0.597	0.611	0.435	**0.972**	0.778	0.736	0.729	0.646
MAE	1.767	4.011	4.153	4.034	5.039	**0.932**	2.703	3.053	3.365	4.054
MAPE	1.453%	3.320%	3.462%	3.357%	4.210%	**0.775%**	2.243%	2.535%	2.799%	3.387%
RMSE	2.239	5.220	5.152	5.070	6.104	**1.353**	3.791	4.172	4.230	4.837

**Table 4:** Results of estimating live weight of pigs using the five features of Area, Per, PBL, PHW and Ecc.

Method	XGBoost	BP	Lasso	LR	MLP	RFR	Stacking	SVR	KRR	Adaboost
$R^{2}$	0.836	0.564	0.603	0.610	0.269	**0.970**	0.776	0.735	0.722	0.621
MAE	2.595	4.120	4.108	4.035	5.570	**0.936**	2.682	3.078	3.437	4.072
MAPE	2.14%	3.43%	3.42%	3.36%	4.66%	**0.78%**	2.21%	2.56%	2.86%	3.40%
RMSE	3.441	5.563	5.121	5.077	6.946	**1.335**	3.791	4.181	4.286	5.00

**Table 5:** The weight estimation results of strategy 2.

Method	XGBoost	BP	Lasso	LR	MLP	RFR	Stacking	SVR	KRR	Adaboost
$R^{2}$	**0.995**	0.606	0.610	0.620	0.516	0.971	0.781	0.741	0.729	0.648
MAE	**0.389**	4.123	4.222	4.173	4.651	0.950	2.594	3.005	3.298	3.957
MAPE	**0.318%**	3.406%	3.526%	3.480%	3.874%	0.790%	2.158%	2.507%	2.757%	3.296%
RMSE	**0.576**	5.102	5.074	5.008	5.652	1.383	3.805	4.135	4.230	4.816

**Table 6:** The weight estimation results of strategy 3.

Method	XGBoost	BP	Lasso	LR	MLP	RFR	Stacking	SVR	KRR	Adaboost
$R^{2}$	0.873	0.654	0.688	0.706	0.540	**0.974**	0.808	0.756	0.754	0.669
MAE	2.173	3.627	3.609	3.480	4.561	**0.895**	2.429	2.870	3.063	3.744
MAPE	1.790%	2.992%	3.017%	2.905%	3.810%	**0.745%**	2.016%	2.392%	2.556%	3.119%
RMSE	3.024	4.777	4.510	4.407	5.513	**1.300**	3.559	4.013	4.030	4.676

**Table 7:** Comparison of our results with those of previous studies.

Research	Variables Source	Approach to Estimation	R^2^	MAE
Jun et al. (2018) [18]	3D image	Fully connected	0.7900	3.15 kg
Femandes et al. (2020) [28]	-	Neural network deep learning	0.8600	3.26 kg
Li et al. (2022) [25]	Point clouds	Ridge regression	0.958	2.96 kg
Kwon et al. (2023) [29]	Point clouds	Deep neural network	0.9532	4.8847
Liu et al. (2024) [30]	Point clouds	MACNN	-	11.181 kg
our	3D image	Mask R-CNN + Ensemble regression model	0.995	0.389 kg

## Data Availability

The data sets presented in this article are not readily available because the data are part of an ongoing study.
